# Supplementary material for: Expanding protected area coverage for migratory birds could improve long-term population trends
Source: Nat Commun. 2025 Feb 20;16:1813. doi: 10.1038/s41467-025-57019-x (PMC11842860; doi:10.1038/s41467-025-57019-x)
Supplement: Supplementary file 1 — Supplementary Information [file 41467_2025_57019_MOESM1_ESM.pdf]

Expanding protected area coverage for migratory birds could improve long-term population trends

Supplementary Table 1. AUC for Spatiotemporal Exploratory Models. Area Under Curve (AUC) values (means over 5-fold cross validation with ± standard errors) for STEMs of species occurrence in a list or pseudo list against habitat, climate, topography and space and time (n = 4,874,228 see Supplementary Data 1 for full list of variables and Supplementary Table 2 for species scientific names).

Species	AUC 5-fold cross-validation
Collared Flycatcher	0.954 ± 0.021
Golden Oriole	0.952 ± 0.009
Common Nightingale	0.952 ± 0.006
Marsh Warbler	0.944 ± 0.028
Red-backed Shrike	0.933 ± 0.015
Common Cuckoo	0.923 ± 0.010
Common Whitethroat	0.923 ± 0.010
European Pied Flycatcher	0.922 ± 0.023
Common Swift	0.916 ± 0.007
Sedge Warbler	0.914 ± 0.025
Garden Warbler	0.913 ± 0.011
Lesser Whitethroat	0.910 ± 0.021
European Turtle Dove	0.910 ± 0.014
Willow Warbler	0.910 ± 0.011
Wood Warbler	0.908 ± 0.038
Eurasian Hoopoe	0.905 ± 0.006
Barn Swallow	0.890 ± 0.008
Whinchat	0.876 ± 0.024
Ortolan Bunting	0.874 ± 0.025
Common Sand Martin	0.874 ± 0.018
Yellow Wagtail	0.869 ± 0.019
Eurasian Blackcap	0.869 ± 0.011
Eurasian Wryneck	0.868 ± 0.033
Tree Pipit	0.868 ± 0.024
Common Redstart	0.868 ± 0.021
Northern House Martin	0.866 ± 0.007
Common Wheatear	0.855 ± 0.017
Ring Ouzel	0.851 ± 0.029
Common Chiffchaff	0.850 ± 0.010
European Nightjar	0.829 ± 0.047

Supplementary Methods 1. Exploring yearly variation

Our EuroBirdPortal (EBP) data spanned 10 years with more data towards the end of this time period than the beginning (10 times more records from 2019 than from 2010). Therefore, predictions made for early years in this time frame may be less reliable. In the main text, we made predictions with year as 2018, as a compromise between choosing a year with a high number of records (2018 has the second highest number of records) and a year closer to the range of years used for the climate variables (see Supplementary Data 1). We chose a more recent year because we felt this would be more likely to reflect the current situation. However, there will be yearly variability in the timing of species distributions. To better understand how this might influence our results we also made predictions for 2011 and 2015 using the same parameters as for the 2018 predictions reported in the main text. Using these predictions for 2011 and 2015, we then repeated the procedure described in the methods to determine the percentage cover of protected areas for each species predicted distribution in each week where the summed occurrence was greater than or equal to 25% of the maximum summed occurrence. We then compared the difference in species weekly coverage by protected areas (Supplementary Figure 1). From this we found that most year variation occurred during passage (Supplementary Figure 1). The vast majority of differences in protected area cover between years (92.1 %) were less than 2%, with very few (1.1%) of differences between years greater than 4% (Supplementary Figure 2).

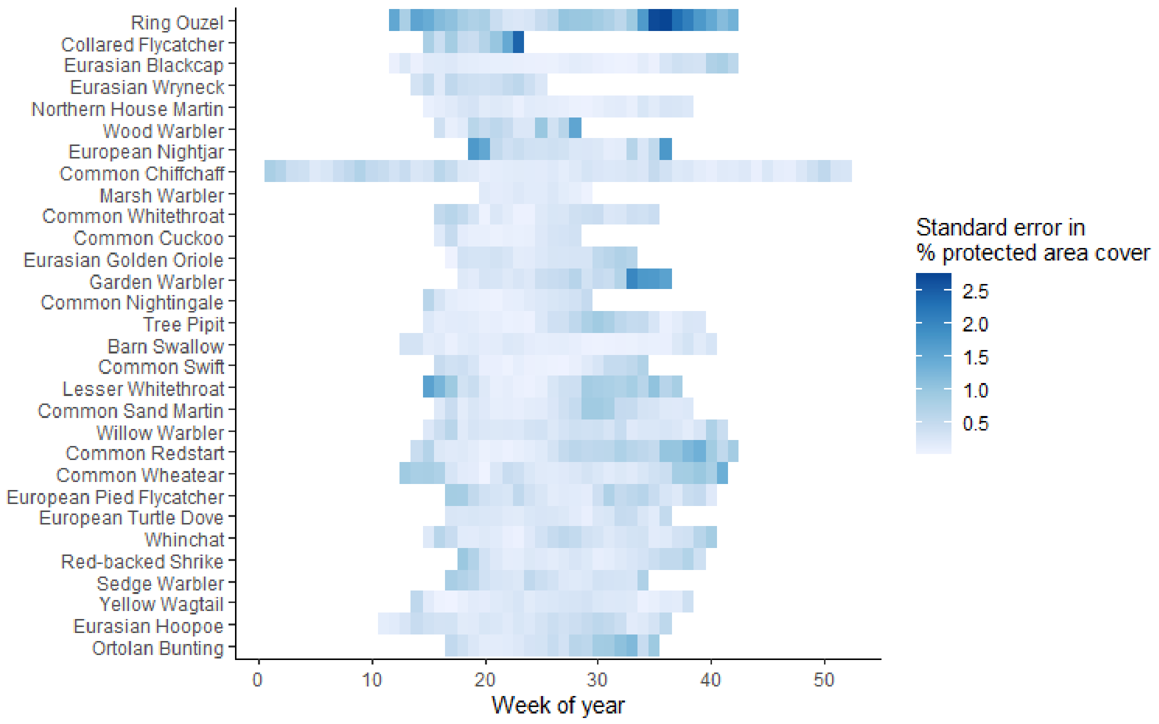

Supplementary Figure 1. Standard error in percentage protected area cover between three years. The standard error in percentage protected area cover between three years (2011, 2015 and 2018). We only included weeks where a species summed occurrence was greater than or equal to 25% the species maximum summed occurrence in at least 2 years (n = 667). The dataset used in this Figure is available in Source Data.

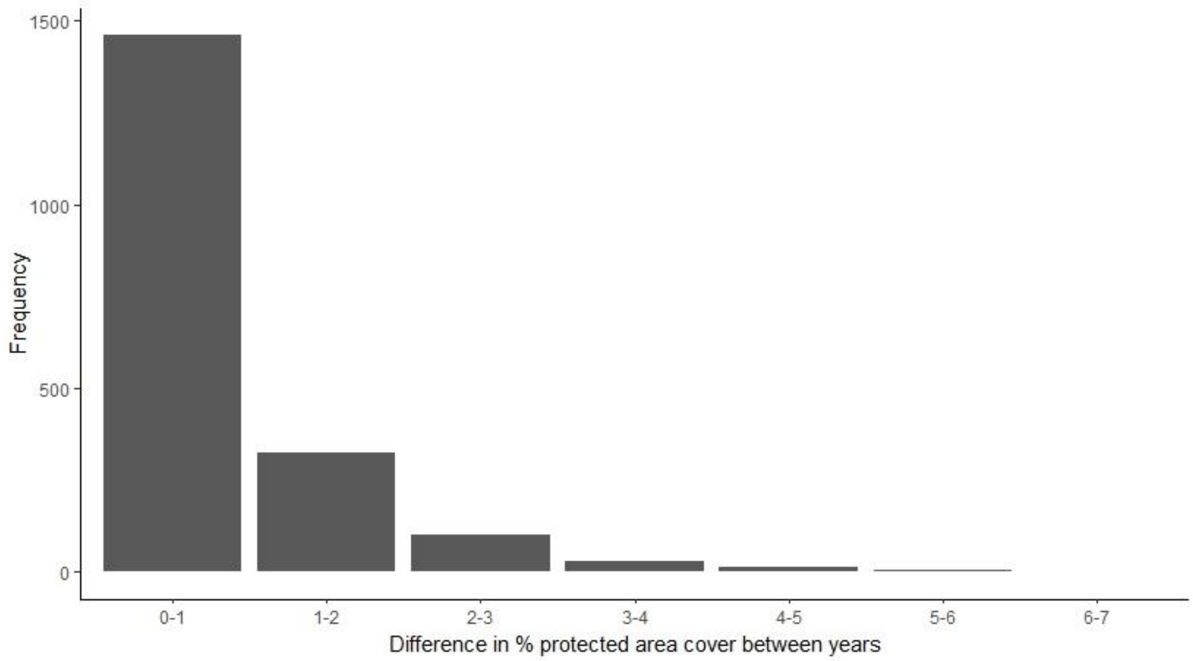

Supplementary Figure 2. Histogram of differences in percentage protected area cover between years. Histogram of the distribution of differences in percentage protected area cover across species between three years (2011, 2015, 2018, n =1941). The dataset used in this Figure is available in Source Data.

Supplementary Methods 2. Sensitivity analysis on the effect of species clumping in or avoiding protected areas.

To explore the sensitivity of our results to our use of summed species occurrences as a metric for assessing protected area coverage, we tested two alternative approaches using binary thresholds to classify a 10 km square as protected or not. In our first scenario, we classed the entire 10 km square as protected if at least 10% of it was protected[1], and the second, if at least 50% of the square was protected[2]. Squares that did not meet our thresholds were classed as unprotected. These scenarios are designed to represent the extremes of possibility. Realistically, we would expect protection to have a wider-benefit beyond the area of land protected. Sanderson et al.[3] found that SSSI and Natura 2000 protected areas can have benefits up to 5 km away on birds of conservation concern. The relationship between protected area size and effectiveness is also unclear, although previous work does suggest some benefits of coverage as small as 10%[1]. Further, we recognise that some protected areas may have management that is counter to the protection of certain bird species (e.g. clearing areas of woodland for the conservation of open country species or habitats will be detrimental to migratory woodland passerines), and that the precise benefits of protection will vary between species depending on their habitat and management requirements[1]. The continental and multi-species scale of our analysis is therefore necessarily coarser in terms of resolution and assumptions than would ideally be undertaken for individual species.

The two scenarios for classifying protected areas naturally have a significant impact on the overall amount of protection across Europe with 38.4% and 16.9 % of squares classed as protected under the 10% and 50% scenarios respectively. As a result, the number of species considered adequately protected also varies (Supplementary Figure 3). Under the 10% scenario only two species appear inadequately protected for some of their time in Europe under our 17% target (Ortolan Bunting and Common Redstart) and ten (Red-backed Shrike, Eurasian Hoopoe, Lesser Whitethroat, Ortolan Bunting, European Pied Flycatcher, Common Redstart, Common Wheatear, Whinchat, Willow Warbler and Yellow Wagtail) under the 30% target. Under the 50% scenario, we found that 24 species were inadequately protected under our 17% target (all except Ring Ouzel, Collared

Flycatcher, Eurasian Blackcap, Eurasian Wryneck, House Martin and Wood Warbler) and all species were inadequately protected under the 30% protected area cover target.

From this analysis we can see that the absolute percentages of protected area cover are sensitive to which binary threshold is selected, though the difference in coverage between species and temporally is qualitatively similar for all 3 methods. Therefore, to avoid commission and omission errors[4,5,6] we follow Araújo et al.[4] in the main text and assume a species is equally distributed throughout a 10 km square over the course of a week.

a)

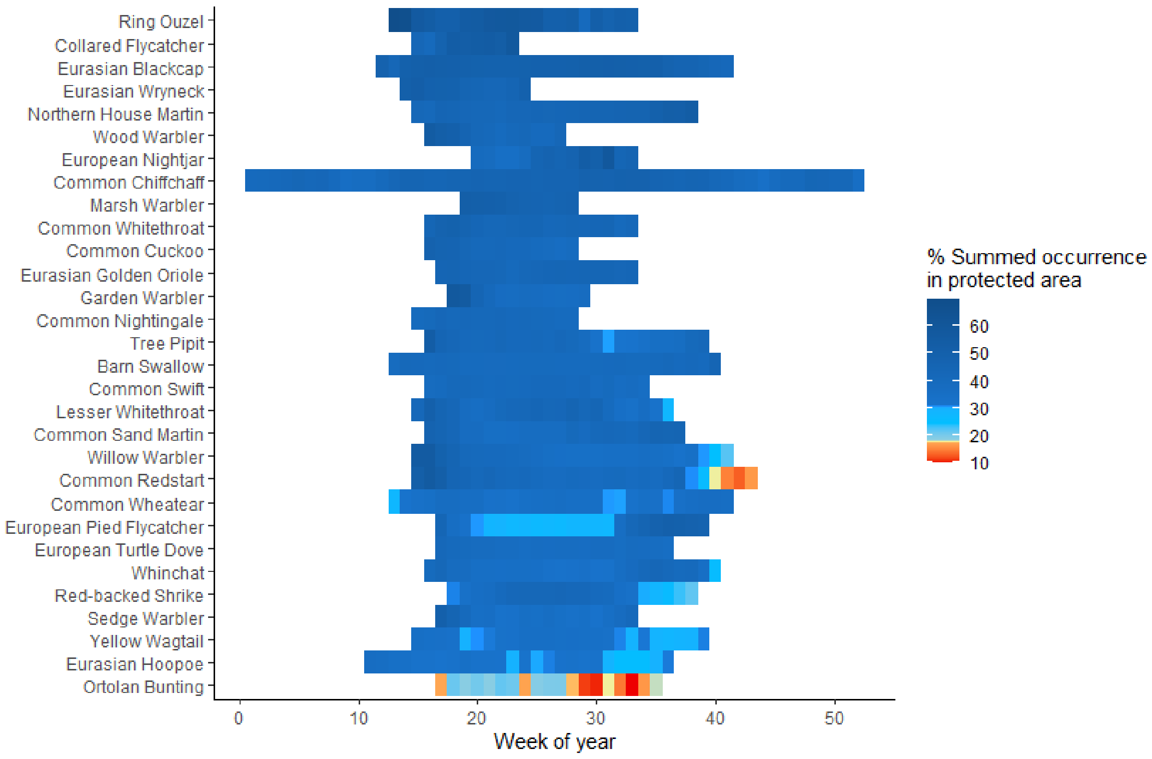

b)

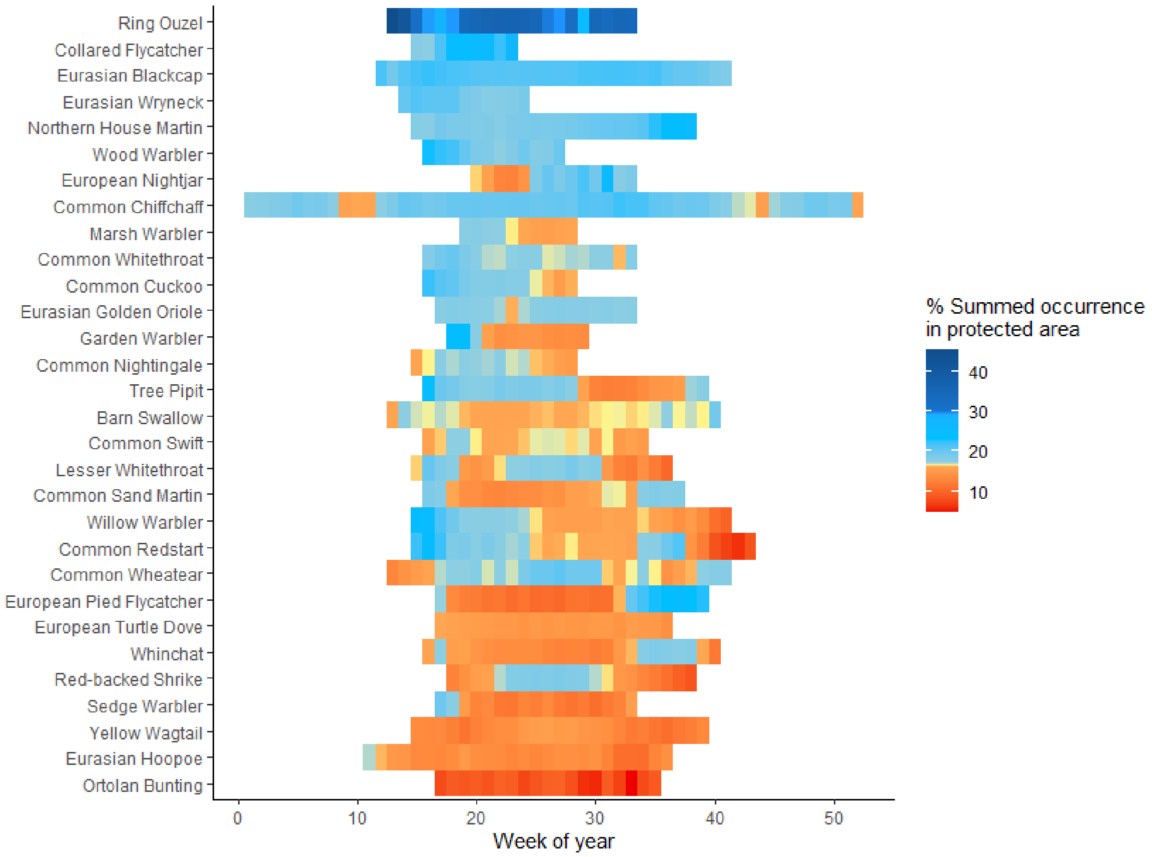

Supplementary Figure 3. The weekly percentage cover of protected areas under two threshold assumptions. The weekly percentage of summed occurrence in protected areas per species (shown by colour ramp) assuming that a) if 10% or more of a 10 km square is protected this equates to 100% protected area cover, but less than 10% is equivalent to 0% protected area cover (n = 633) and b) if 50% or more of a 10 km square is protected this equates to 100% protected area cover, less than this is equivalent to 0% protected area cover (n =633). To facilitate comparisons, the same colour ramp is used for both panels (i.e. the same shade of orange is used to denote a 15% summed occurrence in protected areas).

Supplementary Table 2. The species included in this analysis. We focused purely on European-African migrant passerines and near-passerines and were restricted to species covered by EuroBirdPortal.

English Name	Scientific Name	Species code
Barn Swallow	*Hirundo rustica*	SL
Collared Flycatcher	*Ficedula albicollis*	FA
Common Chiffchaff	*Phylloscopus collybita*	CC
Common Cuckoo	*Cuculus canorus*	CK
Common Nightingale	*Luscinia megarhynchos*	N.
Common Redstart	*Phoenicurus phoenicurus*	RT
Common Sand Martin	*Riparia riparia*	SM
Common Swift	*Apus apus*	SI
Common Wheatear	*Oenanthe Oenanthe*	W.
Common Whitethroat	*Sylvia communis*	WH
Eurasian Blackcap	*Sylvia atricapilla*	BC
Eurasian Hoopoe	*Upupa epops*	HP
Eurasian Wryneck	*Jynx torquilla*	WY
European Nightjar	*Caprimulgus europaeus*	NJ
European Pied Flycatcher	*Ficedula hypoleuca*	PF
European Turtle Dove	*Streptopelia turtur*	TD
Garden Warbler	*Sylvia borin*	GW
Golden Oriole	*Oriolus oriolus*	OL
Lesser whitethroat	*Sylvia curruca*	LW
Marsh Warbler	*Acrocephalus palustris*	MW
Northern House Martin	*Delichon urbicum*	HM
Ortolan Bunting	*Emberiza hortulana*	OB
Red-backed Shrike	*Lanius collurio*	ED
Ring Ouzel	*Turdus torquatus*	RZ
Sedge Warbler	*Acrocephalus schoenobaenus*	SW
Tree Pipit	*Anthus trivialis*	TP
Whinchat	*Saxicola rubetra*	WC
Willow Warbler	*Phylloscopus trochilus*	WW
Wood Warbler	*Phylloscopus sibilatrix*	WO
Yellow Wagtail	*Motacilla flava*	YW

Supplementary Methods 3: Recording schemes

Portals

Artportalen (https://artportalen.se/)

Artsobservasjoner (https://artsobservasjoner.no/)

Aves-Symfony (http://aves.vtaky.sk/en/zoology)

Birds.cz (https://birds.cz/avif/)

BirdTrack (https://app.bto.org/birdtrack/login/login.jsp)

Dabasdati (http://dabasdati.lv/)

DOFbasen (http://www.dofbasen.dk/)

eBird (https://ebird.org/)

Ornitho (https://data.biolovision.net/)

MAP (http://map.mme.hu/)

Observation.org (https://observation.org/)

OpenBirdMaps (http://www.openbirdmaps.ro/)

OrnitoData (http://pasaridinromania.sor.ro/ornitodata)

Plutof (https://plutof.ut.ee/)

SmartBirds (http://www.smartbirds.org/)

Sovon Live Atlas (https://www.liveatlas.nl/)

Tiira (http://tiira.fi/)

Trektellen (https://www.trektellen.org/)

Supplementary Table 3. Cover of habitat types in EuroBirdPortal lists. Comparing the percentage cover of 10 core habitat types in the EuroBirdPortal (EBP) data (n =4,874,228) versus over all 10 km squares and weeks included in the geographical extent of the EBP data (n = 5,824,780) see Supplementary Figure 4). The habitat types are the core ones used in the species specific Spatiotemporal Exploratory Models.

Habitat	EBP	Wider Landscape
Mixed Broadleaved and Coniferous Forest	3.0%	6.7%
Coniferous Forest	10.3%	17.1%
Broadleaved Forest	6.9%	13.1%
Grassland	16.2%	9%
Herbaceous Cover	29.9%	14.3%
Irrigated Cropland	1.1%	1.3%
Mosaic Cropland-natural Vegetation	6.9%	8.4%
Mosaic Natural Vegetation	6.8%	6.2%
Rainfed Cropland	7.3%	18.6%
Urban	10.2%	2%
Wetlands	1.2%	3.2%

a)

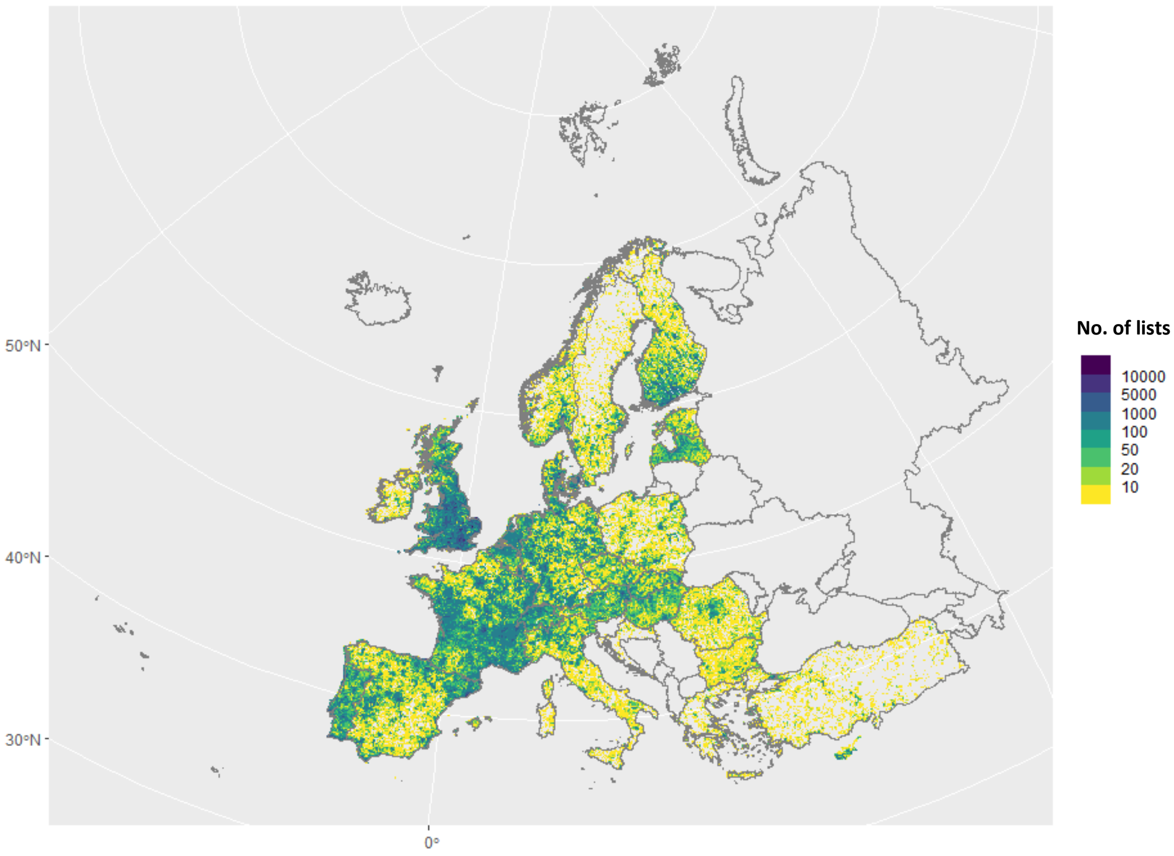

b)_

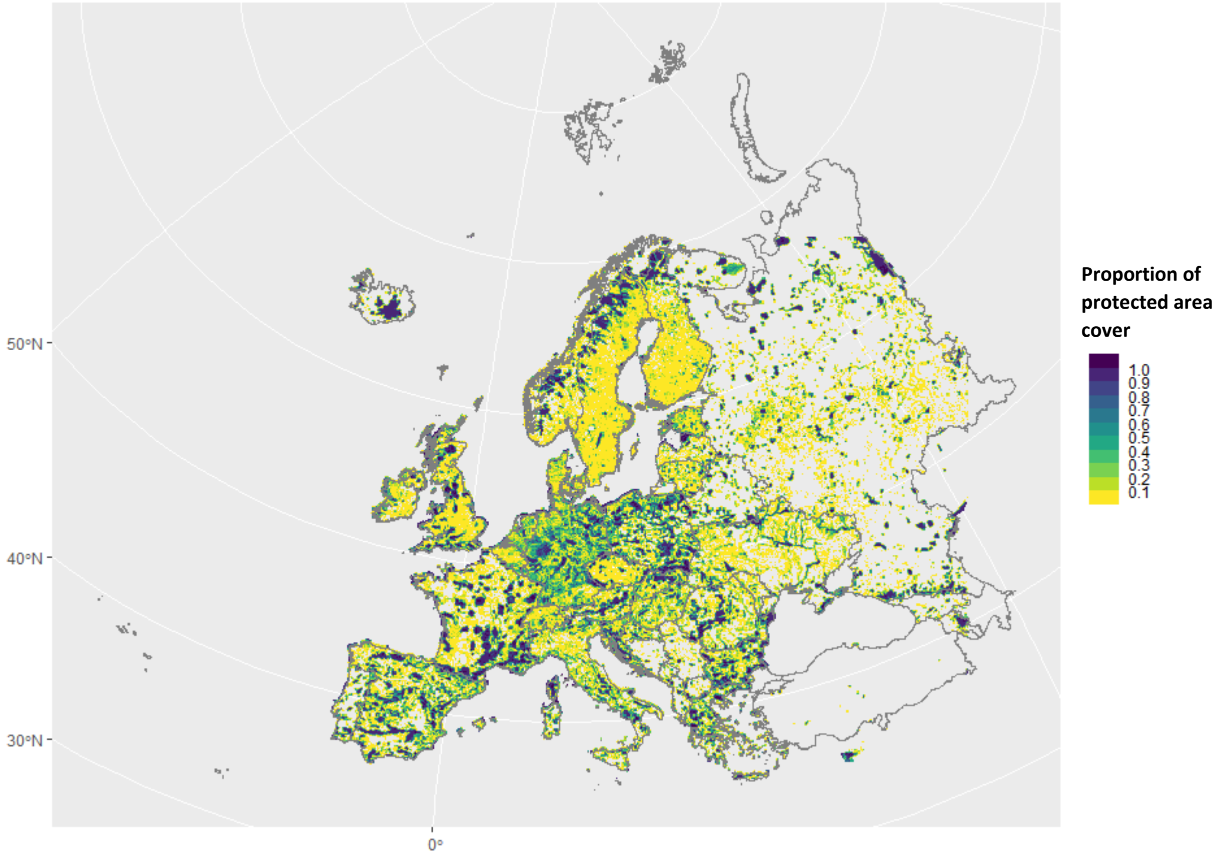

Supplementary Figure 4. **Maps of distribution of EuroBirdPortal lists and protected areas**. 10 km square resolution maps of a) the distribution of the EuroBirdPortal lists used in this analysis, b) the distribution of protected areas in Europe.

Supplementary Methods 4. Statistical Packages

All analysis was carried out in R version 4.2.0. For data manipulation we used data.table[7] (v1.16.0), dplyr[8] (v1.1.4) and tidyr[9] (v1.3.1). Our background maps for Supplementary Figure 4 were created using rnaturalearth[10] (v0.3.4), plotting was done in ggplot2[11] (v3.5.1). Our model to generate pseudo complete lists from casual records used a random forest model from the R ranger package[12] (v0.13.1). Our STEM models used the packages gbm[13] (v2.1.9) and rpart[14] (v4.1.16). To validate our STEMs we used the blockCV[15] (v2.1.4) R package to undertake environmental blocking and the pROC[16] (v1.18.0) R package to generate the AUC statistic. For our Linear Mixed Models we used the R package glmmTMB[17] (v1.1.3).

Supplementary References

1. Barnes, A. E., Davies, J. G., Martay, B., Boersch-Supan, P. H., Harris, S. J., Noble, D. G., ... & Robinson, R. A. Rare and declining bird species benefit most from designating protected areas for conservation in the UK. Nature Ecology & Evolution, 7(1), 92-101. (2023).
2. Runge, C. A., Tulloch, A. I., Possingham, H. P., Tulloch, V. J., & Fuller, R. A. Incorporating dynamic distributions into spatial prioritization. Diversity and Distributions, 22(3), 332-343. (2016).
3. Sanderson, F. J., Wilson, J. D., Franks, S. E., & Buchanan, G. M. Benefits of protected area networks for breeding bird populations and communities. Animal Conservation, 26(3), 279-289. (2023).
4. Araújo, M. B., Alagador, D., Cabeza, M., Nogués-Bravo, D., & Thuiller, W. Climate change threatens European conservation areas. *Ecology letters*, *14*(5), 484-492. (2011).
5. Araújo, M. B. Matching species with reserves–uncertainties from using data at different resolutions. *Biological Conservation*, *118*(4), 533-538. (2004).
6. Alagador, D., Martins, M. J., Cerdeira, J. O., Cabeza, M., & Araújo, M. B. A probability-based approach to match species with reserves when data are at different resolutions. *Biological Conservation*, *144*(2), 811-820. (2011).
7. Barrett T, Dowle M, Srinivasan A, Gorecki J, Chirico M, Hocking T, Schwendinger B (2024). data.table: Extension of `data.frame`. R package version 1.16.0 <https://CRAN.R project.org/package=data.table>.
8. Wickham H, François R, Henry L, Müller K, Vaughan D (2023). dplyr: A Grammar of Data Manipulation. R package version 1.1.4, <https://CRAN.R-project.org/package=dplyr>.
9. Wickham H, Vaughan D, Girlich M (2024). tidyr: Tidy Messy Data. R package version 1.3.1, <https://CRAN.R-project.org/package=tidyr>.
10. Massicotte P, South A (2023). rnaturalearth: World Map Data from Natural Earth. R package version 0.3.4, <https://CRAN.R-project.org/package=rnaturalearth>.

11. Wickham., H. ggplot2: Elegant Graphics for Data Analysis. Springer-Verlag New York, 2016.
12. Wright, M. N., Ziegler, A. ranger: A Fast Implementation of Random Forests for High Dimensional Data in C++ and R. Journal of Statistical Software, 77(1), 1-17. doi:10.18637/jss.v077.i01 (2017).
13. Greg R, Developers G (2024). gbm: Generalized Boosted Regression Models. R package version 2.1.9, <https://CRAN.R-project.org/package=gbm>.
14. Therneau T, Atkinson B (2022). rpart: Recursive Partitioning and Regression Trees. R package version 4.1.16, <https://CRAN.R-project.org/package=rpart>.
15. Valavi, R., Elith, J., Lahoz-Monfort, J. J., & Guillera-Arroita, G. blockCV: An r package for generating spatially or environmentally separated folds for k-fold cross-validation of species distribution models. Biorxiv, 357798. (2018).
16. Robin, X., Turck, N., Hainard, A., Tiberti, N., Lisacek, F., Sanchez, J-C. and Müller, M. pROC: an open-source package for R and S+ to analyze and compare ROC curves. BMC Bioinformatics, 12, p. 77. DOI: 10.1186/1471-2105-12-77 http://www.biomedcentral.com/1471-2105/12/77/ (2011).
17. Brooks M.E, Kristensen K., van Benthem K.J., Magnusson A., Berg C.W., Nielsen A., Skaug H.J., Maechler M., Bolker B.M. glmmTMB Balances Speed and Flexibility Among Packages for Zero-inflated Generalized Linear Mixed Modeling. The R Journal, 9(2), 378–400. https://journal.r-project.org/archive/2017/RJ-2017-066/index.html. (2017).
