# Supplementary material for: Expanding protected area coverage for migratory birds could improve long-term population trends
Source: Nat Commun. 2025 Feb 20;16:1813. doi: 10.1038/s41467-025-57019-x (PMC11842860; doi:10.1038/s41467-025-57019-x)
Supplement: Supplementary file 3 — Reporting Summary [file 41467_2025_57019_MOESM3_ESM.pdf]

nature portfolio

Corresponding author(s): Jennifer Border
Last updated by author(s): Apr 11, 2024

Reporting Summary

Nature Portfolio wishes to improve the reproducibility of the work that we publish. This form provides structure for consistency and transparency in reporting. For further information on Nature Portfolio policies, see our Editorial Policies and the Editorial Policy Checklist.

Statistics

For all statistical analyses, confirm that the following items are present in the figure legend, table legend, main text, or Methods section.

n/a	Confirmed	
☐	☒	The exact sample size (*n*) for each experimental group/condition, given as a discrete number and unit of measurement
☐	☒	A statement on whether measurements were taken from distinct samples or whether the same sample was measured repeatedly
☐	☒	The statistical test(s) used AND whether they are one- or two-sided<br>*Only common tests should be described solely by name; describe more complex techniques in the Methods section.*
☐	☒	A description of all covariates tested
☐	☒	A description of any assumptions or corrections, such as tests of normality and adjustment for multiple comparisons
☐	☒	A full description of the statistical parameters including central tendency (e.g. means) or other basic estimates (e.g. regression coefficient) AND variation (e.g. standard deviation) or associated estimates of uncertainty (e.g. confidence intervals)
☐	☒	For null hypothesis testing, the test statistic (e.g. *F*, *t*, *r*) with confidence intervals, effect sizes, degrees of freedom and *P* value noted<br>*Give P values as exact values whenever suitable.*
☒	☐	For Bayesian analysis, information on the choice of priors and Markov chain Monte Carlo settings
☒	☐	For hierarchical and complex designs, identification of the appropriate level for tests and full reporting of outcomes
☒	☐	Estimates of effect sizes (e.g. Cohen's *d*, Pearson's *r*), indicating how they were calculated

Our web collection on statistics for biologists contains articles on many of the points above.

Software and code

Policy information about availability of computer code

Data collection	No software was used in data collection, we requested the data from EuroBirdPortal and received it as csv files
Data analysis	We analysed the data using R version 4.2.0. All code is available in Zenodo deposit 10.5281/zenodo.10960419. The packages we used were all open source: data.table (v1.16.0), ggplot2 (v3.5.1), ranger (v0.13.1), blockCV (v2.1.4), pROC (v1.18.0), glmmTMB (v1.1.3), dplyr (v1.1.4) , tidyr (v1.3.1), rnaturalearth (v0.3.4), exactextractr (v 0.8.2), sf (v1.0.7), gbm (v2.1.9) , rpart(v4.1.16)

For manuscripts utilizing custom algorithms or software that are central to the research but not yet described in published literature, software must be made available to editors and reviewers. We strongly encourage code deposition in a community repository (e.g. GitHub). See the Nature Portfolio guidelines for submitting code & software for further information.

Data

Policy information about availability of data

All manuscripts must include a data availability statement. This statement should provide the following information, where applicable:

- Accession codes, unique identifiers, or web links for publicly available datasets
- A description of any restrictions on data availability
- For clinical datasets or third party data, please ensure that the statement adheres to our policy

The raw bird occurrence data analysed in this study have been deposited in the EuroBirdPortal database under the data request id: BTO_03_2020. The raw bird occurrence data are available under restricted access to ensure proper use and knowledge of the data. The data belongs to EuroBirdPortal partners, access can be

obtained (for purposes of replicating the analysis) by emailing Gabriel Gargallo on anella@ornitologia.org and Verena Keller on verena.keller@vogelwarte.ch, requests will be responded to within a week and access will be granted for as long as needed. The processed data (predicted occurrences for each species at a 10 km square resolution, the percentage cover of each 10 km square by protected areas, and animated and stationary weekly distribution maps) are available at Zenodo repository (10.5281/zenodo.10960419). The source data for all graphs generated in this study are provided in the Source Data file. The protected area data used in this study are available in the Protected Planet database from www.protectedplanet.net.

Research involving human participants, their data, or biological material

Policy information about studies with human participants or human data. See also policy information about sex, gender (identity/presentation), and sexual orientation and race, ethnicity and racism.

Reporting on sex and gender	N/A
Reporting on race, ethnicity, or other socially relevant groupings	N/A
Population characteristics	N/A
Recruitment	N/A
Ethics oversight	N/A

Note that full information on the approval of the study protocol must also be provided in the manuscript.

Field-specific reporting

Please select the one below that is the best fit for your research. If you are not sure, read the appropriate sections before making your selection.

☐ Life sciences ☐ Behavioural & social sciences ☒ Ecological, evolutionary & environmental sciences

For a reference copy of the document with all sections, see nature.com/documents/nr-reporting-summary-flat.pdf

Ecological, evolutionary & environmental sciences study design

All studies must disclose on these points even when the disclosure is negative.

Study description	Using citizen science data to model 30 Afro-Palearctic migrant bird's European distributions through the year and then determining the percentage of each species summed occurrence in a protected area for each week and whether this matched policy targets.
Research sample	The EuroBirdPortal (EBP) data set from January 1st 2010 to December 31st 2019 (https://eurobirdportal.org/ebp/en/). EBP is a European Bird Census Council (EBCC) project, with 81 partner institutions from 29 different European countries, which collates bird records from 18 different portals and combines them into a single online dataset. This dataset contains bird records submitted by bird watchers at a daily, 10 x 10 km square resolution. Records are submitted either as complete lists where everything a bird watcher detected is recorded or casual records, where an unknown proportion of birds seen or heard are recorded. Casual lists are aggregated for each 10 km square and day. The end dataset used for analysis contained 4,874,228 lists covering 41,670 10 km squares and 3,652 days over 10 years.
Sampling strategy	We used the maximum sample size possible for each species, after excluding data which was unsuitable for inclusion in our models (see data exclusions). In order to make casual records comparable to complete lists we used a random forest model of list length for complete lists against the coordinates of the complete list, month of the year, elevation and habitat. We then used this model to predict the expected length of a list for each 10 km square and removed casual lists below the predicted value. We selected 30 species for this analysis as we were interested in passerine and near passerine Afro-Palearctic migrant birds and at the time of data extraction these were all the passerine and near passerine Afro-Palearctic migrants available in the EBP data.
Data collection	Participant bird watchers in all participating countries go out bird watching to a site of their choice and record either a complete list of every bird they see or hear or a casual record of some of the birds they see or hear. People submit data all year round and surveys may vary extensively in the time spent and the area surveyed.
Timing and spatial scale	This data covers 29 different European countries and spans from 2010 to 2019.
Data exclusions	We excluded EBP records from remote island groups and countries with very little data as we could not make reliable models for these regions (0.065%). We excluded data from organised surveys explicitly targeting non-target species (e.g. seawatches and raptor surveys, 0.7%). A subset of older records (29%) was removed from the dataset, because they were aggregated at the weekly level for each 10-km square, preventing us being able to determine species presence or absence in a list for a particular day.
Reproducibility	N/A not an experiment
Randomization	N/A not an experiment

Blinding | N/A not an experiment

Did the study involve field work? ☐ Yes ☒ No

Reporting for specific materials, systems and methods

We require information from authors about some types of materials, experimental systems and methods used in many studies. Here, indicate whether each material, system or method listed is relevant to your study. If you are not sure if a list item applies to your research, read the appropriate section before selecting a response.

Materials & experimental systems

n/a	Involved in the study
☒	☐ Antibodies
☒	☐ Eukaryotic cell lines
☒	☐ Palaeontology and archaeology
☐	☒ Animals and other organisms
☒	☐ Clinical data
☒	☐ Dual use research of concern
☒	☐ Plants

Methods

n/a	Involved in the study
☒	☐ ChIP-seq
☒	☐ Flow cytometry
☒	☐ MRI-based neuroimaging

Animals and other research organisms

Policy information about studies involving animals; ARRIVE guidelines recommended for reporting animal research, and Sex and Gender in Research

Laboratory animals	N/A none used in this study
Wild animals	The presence of wild birds was observed and recorded.
Reporting on sex	This data was not collected.
Field-collected samples	N/A no field samples collected
Ethics oversight	N/A birds were only observed not caught or handled.

Note that full information on the approval of the study protocol must also be provided in the manuscript.

Plants

Seed stocks	N/A
Novel plant genotypes	N/A
Authentication	N/A
