# Supplementary material for: Expanding protected area coverage for migratory birds could improve long-term population trends
Source: Nat Commun. 2025 Feb 20;16:1813. doi: 10.1038/s41467-025-57019-x (PMC11842860; doi:10.1038/s41467-025-57019-x)
Supplement: Supplementary file 4 — Description of Additional Supplementary Files [file 41467_2025_57019_MOESM4_ESM.pdf]

# Description of Additional Supplementary Files

Supplementary Data 1

Description: The environmental variables used in the SpatioTemporal Exploratory Models (STEM). The environmental variables used in the species STEM with details on the unit measured and the source.
